# Correlation between intracranial pressure and pulsatility index measured by transcranial Doppler in children with severe trauma brain injury

**DOI:** 10.1186/cc14529

**Published:** 2015-03-16

**Authors:** H Bouguetof, M Negadi, K El Halimi, D Boumendil, Z Chentouf Mentouri

**Affiliations:** 1 University Ahmed Benbella Oran 1, Oran, Algeria

## Introduction

This study was designed to see whether there is a correlation between the transcranial Doppler (TCD) parameters and the CCP and intracranial pressure (ICP) monitoring during the cerebral hemodynamic changes and to evaluate ICP indirectly by TCD.

## Methods

A prospective and descriptive study conducted in our PICU from June 2011 to December 2013. We investigated 51 children with severe trauma brain injury (TBI). The TCD measurements were routinely performed bilaterally on the middle cerebral artery parallel to the ICP registration. The ICP and CPP data were correlated to PI and the correlation coefficient calculated. To control the linear correlation, the residuals were tested for normal distribution around the regression line.

## Results

ICP registrations were made parallel with all TCD measurements in 51 patients. Intraparenchymal ICP monitoring was inserted within the first 3 hours after trauma and there was no complication (infections, intracranial hemorrhage, or technical failure) related to invasive ICP monitoring. Increased ICP was noted upon transducer insertion in 38 children with male prevalence (10 girls, 28 boys). Median GCS was 6, indicating the magnitude of injury in this group of patients. The overall results of the 38 patients showed a strong correlation between the ICP and PI and during outbursts of ICP with a correlation coefficient of *r *= 0.89 (ICP >20 mmHg) and *r *= 0.90 (ICP <20 mmHg). The relation between ICP and PI was estimated by the linear regression equation: ICP = 22,299 × PI - 10,705 (ICP >20 mmHg) and ICP = 38,592 × PI - 16,972 (ICP <20 mmHg). The CPP and PI were correlated significantly during the changes in intracranial pressure. However, a better correlation was found when ICP >20 mmHg and PPC <50 mmHg (PI was 2.4 ± 0.89 when CPP was 35.96 ± 4.48 with a correlation coefficient of Pearson *r *= 0.80) than when ICP <20 mmHg and CPP >50 mmHg (PI was 0.78 ± 0.14 when CPP was 57.11 ± 9.62 with a correlation coefficient of Pearson *r *=0.76).

## Conclusion

The absolute value of the PI is a reliable noninvasive indicator of ICP in pediatric severe TBI. A strong correlation between PI and ICP was demonstrated. Therefore, PI may be of guiding value in the invasive ICP placement decision in the neurointensive care patient when ICP monitoring is not systematically performed. In particular, ICP monitoring remains as grade C in the latest guidelines of management of STBI in children published in 2012.

